# Diet-Induced Low-Grade Metabolic Acidosis and Clinical Outcomes: A Review

**DOI:** 10.3390/nu9060538

**Published:** 2017-05-25

**Authors:** Renata Alves Carnauba, Ana Beatriz Baptistella, Valéria Paschoal, Gilberti Helena Hübscher

**Affiliations:** 1 VP Research Institute, 287, Carlos Petit St, São Paulo 04110-000, Brazil; consultoriacientifica@vponline.com.br (A.B.B.); valeria.paschoal@vponline.com.br (V.P.); 2 Departament of Food Science and Technology, Federal University of Santa Maria, Rio Grande do Sul 97105-900, Brazil; gilberti@gilbertinutri.com.br

**Keywords:** acid-base equilibrium, acidosis, fruits, vegetables, proteins

## Abstract

Low-grade metabolic acidosis is a condition characterized by a slight decrease in blood pH, within the range considered normal, and feeding is one of the main factors that may influence the occurrence of such a condition. The excessive consumption of acid precursor foods (sources of phosphorus and proteins), to the detriment of those precursors of bases (sources of potassium, calcium, and magnesium), leads to acid-base balance volubility. If this condition occurs in a prolonged, chronic way, low-grade metabolic acidosis can become significant and predispose to metabolic imbalances such as kidney stone formation, increased bone resorption, reduced bone mineral density, and the loss of muscle mass, as well as the increased risk of chronic diseases such as type 2 diabetes mellitus, hypertension, and non-alcoholic hepatic steatosis. Considering the increase in the number of studies investigating the influence of diet-induced metabolic acidosis on clinical outcomes, this review gathers the available evidence evaluating the association of this disturbance and metabolic imbalances, as well as related mechanisms. It is necessary to look at the western dietary pattern of most countries and the increasing incidence of non-comunicable diseases for the balance between fruit and vegetable intake and the appropriate supply of protein, mainly from animal sources, so that it does not exceed the daily recommendations.

## 1. Background

Maintaining the acid-base balance is one of the most strongly regulated variables in human physiology. In order to maintain homeostasis, there is a need to balance the ingestion/production of H^+^ and the effective removal of these ions from the body [1,2]. The real concentration of H^+^ ions can be expressed in a logarithmic scale, through pH units. Any change in blood pH, which is maintained within the range of 7.35–7.45, tends to be rapidly controlled by the body’s buffer systems in order to avoid acidaemia (pH lower than 7.35) or alkalosis (pH higher than 7.45) [1].

In parallel to the definitions of acidaemia and alkalosis, it is reported that there may be minimal changes in the value of plasma bicarbonate and blood pH within the range considered normal. This condition, when the pH is balanced at values close to the lower limit (7.35), is called low-grade metabolic acidosis. There are some factors that can lead to low-grade metabolic acidosis, and diet is one of the main factors that may influence the occurrence of this condition [3].

Diet-induced low-grade metabolic acidosis is a condition that has been investigated since the early 1980s, when Kurtz et al. (1983) [4] showed that an increased dietary acid load led to small changes in the acid-base balance (increase in [H^+^] and reduction in [HCOO_3_^−^]). From time to time, other studies have been published focusing on these minimal alterations in the acid-base balance [5,6,7,8], and several terminologies have been used, such as “eubicarbonatemic metabolic acidosis” [9] and “acid retention” [10]. 

Although these changes in the acid-base balance are small, it has been shown that a diet-induced slight decrease in blood pH can have a significant impact on metabolism. In the last 10 years, many studies have been published evaluating the association between the consumption of an acidifying diet and clinical outcomes. Diet-induced metabolic acidosis has been reported to be associated with the impairment of bone metabolism and an increased risk of a number of chronic noncommunicable diseases, such as type 2 diabetes mellitus and hypertension [11,12,13]. Thus, this study aimed to gather the existing evidence regarding the effects of low-grade metabolic acidosis induced by diet on metabolic disturbances and an increased risk of diseases.

## 2. Diet-Induced Acidosis

Diet may contribute to low-grade metabolic acidosis through the ingestion of dietary constituents of non-volatile acids and bases. The nutrients that release acid precursors into the bloodstream are phosphorus and proteins (mostly containing sulfur amino acids, such as cysteine, methionine, and taurine, and cationic amino acids such as lysine and arginine). In addition, sodium chloride (NaCl) intake is reported to be an independent predictor of plasma bicarbonate concentration. Assuming a causal relationship, NaCl may exert approximately 50–100% of the acidosis-producing effect of the dietary acidic load, and is therefore considered a predictor of diet-induced low-grade metabolic acidosis [14]. On the other hand, the nutrients that are precursors of bases are potassium, magnesium, and calcium. Thus, in general, the main foods that release precursors of acids into the bloodstream are mostly of animal origin (except for beans and nuts), and foods that are precursors of bases are mainly those of plant origin [2,3]. 

There are some methods to determine the net endogenous production of acids, such as the quantification of organic and inorganic constituents in urine and faeces, the measurement of 24-hour urine pH, and the evaluation of acid production from dietary constituents [3]. Out of these, the evaluation from dietary constituents has often been used by epidemiological studies evaluating the association between dietary acid load and disease risk (Table 1). The net endogenous acid production (NEAP) is a method developed by Frasseto et al. in 1998 [15] that takes into account the amount of protein and potassium, respectively and considers the main nutrients associated with the release of acids and bases. The potential renal acid load (PRAL), a calculation developed by Remer & Manz (1994) [16], estimates the intestinal absorption rates of the contributing nutrients, the ionic balance of calcium, magnesium, and potassium, and the dissociation of phosphate at pH 7.4. The more negative the PRAL, the more alkalizing the food is. In addition, PRAL can be added to the estimated excretion value of organic acids, a calculation developed by Sebastian (2002) [7].

Thus, from the estimation of the net endogenous production of acids according to the dietary constituents, it is possible to characterize the foods according to their ability to release acids and bases in the bloodstream. In Table 2, the values of the acidifying potential of several foods, calculated using PRAL, are presented. As we can see, the foods that contribute most to the release of acids into the bloodstream are meats (beef, pork, or poultry), eggs, beans, and oilseeds, and the foods that contribute most to the release of bases are fruits and vegetables. If there is an excessive consumption of acid precursor foods, to the detriment of those precursors of bases, volubility of the acid-base balance occurs [2,3,17]. If this acid-base balance disorder occurs in a prolonged and chronic way, low-grade metabolic acidosis may become significant and predispose to diseases [18,19,20,21].

## 3. Dietary Acid Load and Clinical Outcomes

**Figure 1:**
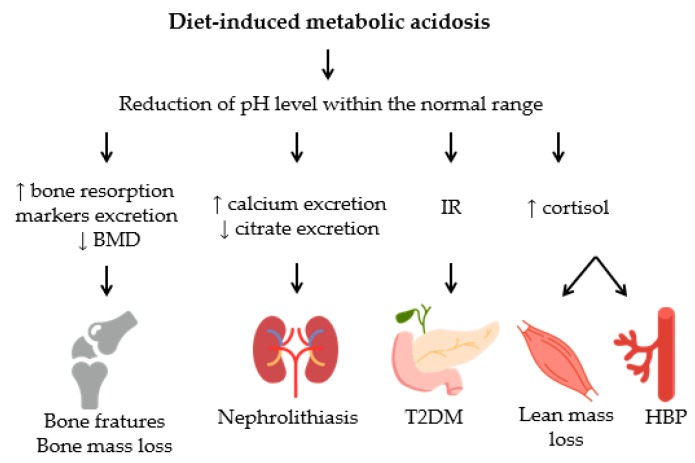
Consequences to health from low-grade metabolic acidosis induced by diet. Dietary-induced low-grade metabolic acidosis may predispose to various disorders including bone metabolism impairment, kidney stone formation, loss of lean mass, increased systemic blood pressure, and risk of type 2 diabetes mellitus. BMD: bone mineral density; IR: insulin resistance; T2DM: type 2 diabetes mellitus; HBP: high blood pressure.

Many observational studies have been conducted to evaluate the association between dietary acid load and disease risk. The negative effects to health from low-grade metabolic acidosis are associated with mineral excretion and an imbalance in hormone secretion, as shown in Figure 1.

### 3.1. Bone Tissue

**Figure 2:**
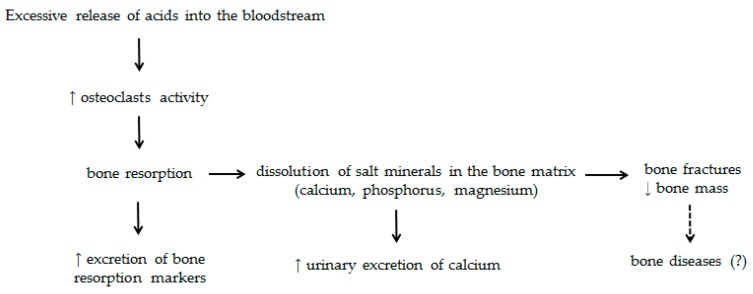
Schematic representation of the excessive release of acids into the bloodstream on bone tissue. The excess of acid release into the bloodstream, in detriment of the release of bases, may predispose to impaired bone metabolism, which involves the increased release of PTH with a consequent increase in osteoclasts activity and bone resorption, reducing the bone tissue mineralization. As a consequence, there may be an increased risk of bone fractures and bone mass reduction.

Some mechanisms have been proposed to explain the influence of an increased dietary acid load on bone metabolism (Figure 2). The slight reduction of the extracellular fluid pH suppresses the activity of osteoblasts and decreases the gene expression of specific matrix proteins and alkaline phosphatase activity. In addition, low-grade metabolic acidosis has been associated with osteoclast activity and increased urinary calcium excretion without increased intestinal calcium absorption, resulting in the depletion of bone calcium [22,23]. Net acid excretion (NAE), a predictor of the acidifying potential of the diet, is shown to be associated with increased serum levels of parathyroid hormone (PTH) and the urinary excretion of calcium and *N*-telopeptide, an important marker of bone resorption [20]. In the study conducted by Buclin et al. (2001) [11], the ingestion of an acidogenic diet for four days caused an increase in the urinary excretion of calcium and *C*-telopeptide of 74% and 19%, respectively, compared to the intake of an alkalizing diet for the same period. Moreover, the consumption of this dietary pattern was associated with a discrete, but significant, reduction in blood and urinary pH.

It has been suggested that the ingestion of an acidifying diet is associated with the reduction of mineralization and bone mass, and a higher risk of fractures. New et al. (2004) [24] showed that high NEAP values were correlated with a lower bone mineral density of the femur and with a reduction of the hip and spine bone mass in 1056 women, independently of age and menopausal status. Recently, Esche et al. (2016) [25] showed that the net acid excretion was inversely related to the mineral content and the cortical bone area, and was positively associated with the risk of bone fractures in women. 

Corroborating these findings, it has been suggested that a diet with negative PRAL is associated with a reduction in bone turnover. Gunn et al. (2015) [26] showed that the consumption of ≥9 servings of fruits and vegetables per day for 12 weeks (diet with PRAL value of −23 mEq/day) was associated with a reduction in the urinary excretion of procollagen type I N propeptide (bone formation marker) and calcium, in addition to increasing the urine pH. Furthermore, a significant reduction in the excretion of C-terminal telopeptide of type I collagen (bone resorption marker) was observed in participants with osteopenia.

Studies have shown that the deleterious effects of low-grade metabolic acidosis on bone tissue are independent of calcium intake [11,27]. Shariati-Bafghi et al. (2014) [27] reported that, in 151 postmenopausal women, PRAL and NEAP were inversely associated with the bone mineral density—an association that remained significant even in those participants with a calcium intake above 800 mg/day [27]. Recently, Kong et al. (2017) [28] showed in their study with 7187 participants from the Korean National Health and Nutrition Examination Survey (KNHANES) that the dietary intake of potassium was positively associated with a higher mineral density in the lumbar spine, femur, and hip, even in participants with a low dietary calcium intake—a fact that can be attributed to the lower acidifying potential of diets rich in food sources of potassium [28].

Despite the evidence hereby described, when assessing the association between dietary acid load and the risk of bone diseases, such as osteoporosis, the results are inconclusive. The available studies evaluating this association are not controlled for all factors related to the risk and progression of bone tissue diseases, such as family history, estrogen status, and renal function, which may, at least in part, explain the inconsistent results emerging from this association. However, since demineralization and the loss of bone mass are associated with various diseases of bone tissue [29,30,31], it is believed that, in the long term, the excessive release of acids into the bloodstream may have a significant impact on the risk of bone diseases.

Concerning the effect of specific dietary components, many questions are raised about the association between proteins, the main acidogenic component of the diet, and bone health. It has been suggested that hyperproteic diets can cause an increase in urinary calcium excretion [16] and, therefore, it is believed that a high protein intake may increase the risk of bone diseases. In addition, proteins are the main contributor to the acidifying potential of the diet. In contrast to these facts, dietary proteins (mainly animal sources) may exert an anabolic effect on bone turnover by increasing levels of insulin-like growth factor (IGF-1), stimulating intestinal calcium absorption, suppressing PTH action, and improving shape and muscle mass [32,33,34]. Furthermore, bones are formed by the protein matrix, and dietary proteins appear to exert an osteotrophic effect [33,35]. Considering these facts together, it is raised that in the presence of an adequate dietary intake of base-forming nutrients, proteins may exert benefits on the bones [32].

### 3.2. Kidney Stones

The effects of an acidogenic diet on the formation of kidney stones are the clinical outcome with the highest number of published studies. In response to diet-induced low-grade metabolic acidosis, the kidneys perform adaptive responses in an attempt to restore the acid-base balance, and these responses include an increased excretion of calcium and oxalate salts, and reduced citrate excretion. Citrate inhibits the formation and agglomeration of calcium oxalate crystals and, thus, the reduction of its excretion is associated with the formation of a less soluble complex of calcium oxalate [2,36]. In their study involving 187 renal stone-formers, Trinchieri et al. (2006) [36] showed that the acidifying dietary pattern (PRAL value between 16 and 24 mEq/day) was associated with reduced citrate excretion in the urine [36].

The study conducted by Ferraro et al. (2016) [37] evaluated the association between dietary acid load and the risk of kidney stone formation in the participants of Professionals Follow-Up Study, the Nurses ‘Health Study I, and the Nurses’ Health Study II. It was found that NEAP and the animal protein-to-potassium ratio were positively associated with the risk of developing kidney stones (increased risk by 10–41%) [37]. Corroborating this finding, Trinchieri et al. (2013) [18] showed that a diet with PRAL higher than 31 mEq/day was associated with a 2.5-fold higher odds of developing kidney stones, a probability that was significantly reduced with the ingestion of two or more portions of vegetables per day [18]. Similarly, another study showed that individuals in the last tertile of PRAL were 2.9 times more likely to form kidney stones compared to those in the first tertile [38].

### 3.3. Chronic Kidney Disease

Two mechanisms have been proposed to elucidate the associations between dietary acid load and chronic kidney disease (CKD). As the demand for acid elimination rises, there is an increase in endothelin-1, angiotensin II, and aldosterone production. These substances are associated with the reduction of the glomerular filtration rate (GFR) and the stimulation of pro-fibrotic factors, which are associated with renal fibrosis. In addition, in an attempt to neutralize the H^+^ load, an increase in ammonia production occurs in the proximal tubule, which can cause tubular toxicity and renal damage. These processes, when constantly stimulated, are associated with the increased risk and progression of CKD [39,40,41].

In this context, it has been shown that the serum bicarbonate level is an independent predictor of CKD progression. Raphael et al. (2011) [42] showed that higher serum bicarbonate levels within the normal range (20 to 30 mEq/L) are associated with a reduced risk of negative outcomes in patients with CKD, as dialysis, worsening renal function, and death [42]. An analysis from 217 CKD patients showed that disease biomarkers (hypoalbuminuria and hyperkalemia) were associated with low serum bicarbonate levels [43]. In a retrospective cohort of participants with CKD, a low serum bicarbonate level was an independent risk factor for disease progression, mainly in those with a preserved kidney function [44].

Population studies have reported associations between net acid excretion, PRAL, and NEAP, and the increased risk, progression, and prevalence of CKD. Banerjee et al. (2014) [40] showed that, in 12,293 adult participants in the National Health and Nutrition Examination Survey, the estimated net acid excretion (eNAE) was inversely associated with the estimated GFR (eGFR) and positively associated with albuminuria, markers of CKD [40]. In a cohort study of participants of the Tehran Lipid and Glucose Study, patients in the last quartile of PRAL had a 1.42 higher odds of CKD, independently of age, body mass index, smoking status, and other confounding factors [45]. The Atherosclerosis Risk in Communities Study results showed an association between a higher dietary acid load and CKD (adjusted hazard ratio: 1.14, highest quartile of NEAP compared with the lowest quartile), even after adjusting for confounding variables, including the presence of diabetes and hypertension [46].

Similarly, the data of observational studies support the hypothesis that increasing the dietary alkali load may reduce the risk of CKD. Wesson & Simoni (2010) [47] showed in their study with mice that the dietary alkali slowed the rate of decline in GFR when compared to the endothelin and aldosterone receptor antagonist [47]. Another study has showed that the ingestion of base-producing fruits and vegetables was associated with GFR preservation and reduced urinary angiotensinogen in patients with stage 3 chronic kidney disease [48]. Additionally, in high-risk individuals with type 2 diabetes mellitus who participated in the Ongoing Telmisartan Alone and in Combination with Ramipril Global Endpoint Trial (ONTARGET) study, a vegetable consumption of more than five servings per week reduced the risk of CKD (odds ratio: 0.90, highest versus the lowest tertile of vegetable consumption) and the frequent consumption of fruits and fruit juices was associated with a reduced risk of incident microalbuminuria and risk of CKD (odds ratio: 0.91, highest versus the lowest tertile of weekly fruit consumption) [49]. In summary, based on available data, the dietary acid load seems to be an important predictor of CKD and interventions taking into account the frequent consumption of base-producing foods, such fruit and vegetables, should be considered. 

### 3.4. Type 2 Diabetes Mellitus

The mechanism involving the association between dietary acid load and the risk of diabetes mellitus has not yet been fully elucidated, but it is believed that the maintenance of blood pH close to the lower limit of the normal range may lead to a decrease in the uptake of glucose by the muscle, the disruption of insulin binding to its receptor, and the inhibition of the insulin signaling pathway. This may lead to peripheral insulin resistance, the main risk factor for the development of type 2 diabetes mellitus [22]. 

Mandel et al. (2012) [50] showed that reduced levels of plasma bicarbonate were associated with a higher odds of developing diabetes, independently of factors such as BMI and renal function, in 630 women (mean age: 56 years) who were accompanied for 10 years [50]. In the study conducted by Fagherazzi et al. (2014) [12], which included a total of 66,485 women (mean age: 50 years) from the E3N-EP1C cohort, it was found that high PRAL and NEAP values were associated with a higher risk of diabetes mellitus, independently of a family history of diabetes and carbohydrate intake [12].

A cross-sectional study developed by Akter et al. (2016) [51] reported that high PRAL and NEAP values were associated with the presence of insulin resistance (assessed by homeostasis model assessment insulin resistance—HOMA-IR and homeostasis model assessment *β*-cell function—HOMA-B) in 1732 apparently healthy Japanese workers, aged 19–69 years [51]. In another study conducted by the same group of researchers and including 64,660 participants from the Japan Public Health Center-based Prospective Study, a positive association between diet PRAL and the risk of diabetes mellitus was observed in men, but not in women. However, this association was not significant in individuals aged ≥50 years. The authors discuss whether this result is due, at least in part, to the PRAL value of the diet of younger individuals, which was significantly higher compared to individuals aged ≥50 years [52].

### 3.5. Hypertension

The number of studies evaluating the association between dietary acid load and the risk of hypertension has increased in recent years. Through cross-sectional analyses, research has shown that PRAL and NEAP are positively associated with increased values of diastolic blood pressure [13,53] and systolic blood pressure [13] in elderly individuals (*n* = 673, ages 70–71 years) and in young women (*n* = 1136, ages 18–22 years).

In the study conducted by Zhang et al. (2009) [54], data were collected from 87,293 women enrolled in the Nurses’ Health Study II (NHS II) to assess the association between dietary acid load and the risk of hypertension. During the 14 years of follow-up, it was observed that NEAP and the protein-to-potassium ratio were positively associated with the risk of hypertension, independently of factors such as age, a family history of hypertension, and sodium intake. When compared to the participants who were in the first decile of NEAP, those who were in the last decile had an increased risk of hypertension of 23% [54]. In another study, which included 2028 participants from the Japan Epidemiology Collaboration on Occupational Health Study, PRAL and NEAP were positively associated with the increased chance of hypertension; however, when there was adjustment for renal function, this association did not remain significant [55].

Some mechanisms are suggested to justify the association between a high acid diet load and the risk of hypertension. In the presence of low-grade metabolic acidosis, there is an increase in the pituitary stimulus for ACTH synthesis and a consequent production of cortisol and aldosterone [56], which in excess, may induce an increase in blood pressure [57,58]. In addition, the acid-base balance influences mineral homeostasis by regulating the calcium absorption in the kidneys, and it is reported that the increased urinary excretion of this mineral may be associated with an increased blood pressure [59,60]. In addition, NaCl intake, the most known risk factor associated with the etiology of hypertension, is an independent predictor of diet-induced low-grade metabolic acidosis [14].

### 3.6. Non-Alcoholic Hepatic Steatosis

Recently, Chan et al. (2015) [61] evidenced a positive association between the acidogenic diet and the risk of non-alcoholic hepatic steatosis (NASH) in 793 Chinese individuals aged 19–72 years. An increase of 1.32 in the odds of developing NASH, independently of the intake of fiber, saturated fatty acids, carbohydrates, and proteins—dietary constituents known to influence the risk of NASH–was observed for each 20 mEq/day of NEAP [61]. Although this is the only available study evaluating this association, it can be suggested that the influence of diet on the acid-base balance is a factor that contributes to the development of this disease.

The mechanisms by which an acidogenic diet may influence the pathogenesis of NASH are not fully understood. Taking into account that the slight reduction in plasma pH caused by an acidifying diet is associated with insulin resistance, the consequent hyperglycemia and increased inflammation could contribute to hepatic insulin resistance, which is related to the increase in the availability of free fatty acids, and is a risk factor for the development of the disease [62].

### 3.7. Lean Body Mass Loss

The influence of a diet with acidifying potential on muscle mass has been studied. Evidence indicates that a high dietary intake of potassium is associated with the better preservation of muscle mass in elderly individuals [63]. Chan et al. (2015) [64] evaluated the association between NEAP and longitudinal changes in appendicular muscle mass (AMM) in 3122 elderly subjects. A positive association between NEAP and the loss of AMM was found during the study follow-up period [64]. Similarly, another study showed that a diet with alkalinizing potential (negative PRAL) was associated with a better fat-free mass index in 2689 women aged 18–79 years, independently of factors such as age and physical activity [65].

The mechanism that surrounds the association between a high dietary acid load and the loss of muscle mass involves the effect of low-grade metabolic acidosis on the stimulation of the proteolysis pathways. This stimulus can be triggered by an increased production of glucocorticoids, such as cortisol, which stimulates the degradation of amino acids for release into the bloodstream. Glutamine is essential for the process of tubular ammoniagenesis, important for the elimination of the body’s hydrogen ions [66,67].

### 3.8. Mortality 

As described above, an increased dietary acid load is associated with an increased risk and progression of non-comunicable diseases. In this sense, researchers have investigated whether a higher dietary acid load can be considered a predictor for the prevalence of mortality. In a retrospective cohort study including 31,590 adults, reduced serum bicarbonate levels were inversely associated with all-cause mortality (adjusted hazard ratio: 1.460, lowest quartile of serum bicarbonate compared with the highest quartile). When analyses for cancer mortality and cardiovascular mortality were performed, higher rates were found: compared with the highest quartile, the adjusted hazard ratio of the lowest quartile of serum bicarbonate was 1.604 and 2.647, respectively. Further, acidic (pH ≤ 5.5) and neutral (pH 6.0–7.5) urine pH were associated with higher all-cause mortality (adjusted hazard ratio: 2550), compared to alkaline urine pH (pH ≥ 8.0) [68].

Using data from two large cohorts, Xu et al. (2016) [69] evaluated the association between dietary acid load (assessed by PRAL), and cardiovascular and all-cause mortality in 81,697 individuals who were followed up for 15 years. An association between high PRAL values and higher mortality rates was found. However, this association was weak (compared to neutral PRAL, the HR for all-cause mortality for the 90th percentile of PRAL were 1.03 in women and 1.04 in men, and for cardiovascular disease mortality were HR 1.06 in women and HR 1.06 in men) [69]. 

Despite these evidences showing an association between diet-induced low-grade metabolic acidosis, and all-cause and cause-specific mortality, further studies are needed to confirm these associations, as well as the causality in this relationship.

## 4. Discussion

Several studies have indicated the influence of low-grade metabolic acidosis on health outcomes, and diet is one of the factors that directly influence this condition [70]. This article provides an overview of the currently available evidence regarding the health effects of low-grade metabolic acidosis induced by diet and metabolic disorders. There are several methods for assessing dietary acid load; however, dietary constituent estimation has often been used by epidemiological studies to assess the association between dietary acid load and disease risk. This estimate is mainly calculated through the NEAP and PRAL formulas, which are simple and validated methods to estimate the dietary acid load from diet-composition data. 

Studies have shown that diets with high values of NEAP and PRAL may predispose to several metabolic damages, such as the stimulation of bone resorption associated with a decrease in bone mineral density and bone mass, leading to a higher risk of fractures. There are some interventional and observational studies showing that increasing fruit and vegetable consumption is associated with better bone outcomes, such as reduced reabsorption markers excretion, an increased bone mineral content, and lower fractures and osteoporosis risk [27,71,72,73]. Furthermore, other studies have reported that alkalinizing supplementation (including potassium citrate or potassium bicarbonate) can attenuate the deleteriuous effects of low-grade metabolic acidosis on bone tissue, as demonstrated by Dawson-Hughes et al. (2009 and 2015) [74,75] and Moseley et al. (2013) [76]. However, the effects promoted by alkalinizing supplementation are acute and there must be a dietary pattern change to reduce the risk of negative bone outcomes, and the frequent consumption of base-producing foods should be considered. 

The excessive release of acids into the bloodstream is associated with a higher chance of developing kidney stones, a consequence of the increased urinary excretion of calcium and oxalate salts and reduced citrate excretion (which is associated with an inhibitory effect on the stone formation) [19,77]. As the demand for acid elimination increases, the kidney plays other adaptive responses that may culminate in an increased risk of CKD. As previously mentioned, a diet rich in fruits and vegetable might reduce the formation of kidney stones [18] and CKD risk [27,29]. Since low serum bicarbonate levels are an independent predictor of CKD progression, the high consumption of fruit and vegetables is also associated with a reduced progression of early CKD. Therefore, in renal stone-formers and patients with CKD, the dietary intake of fruits and vegetables should be considered to neutralize the acid retention and to ameliorate metabolic acidosis. 

Current evidence suggests that diet-induced metabolic acidosis is a risk factor for type 2 diabetes mellitus. Some studies have shown that a slight reduction in extracellular pH decreases the beta cell response and leads to a disruption of insulin binding to its receptor. This may lead to peripheral insulin resistance, the main risk factor for the development of type 2 diabetes mellitus [78,79,80]. In addition to high PRAL and NEAP values, other markers of metabolic acidosis have been associated with insulin resistance, such as plasma bicarbonate reduction, an increased anion gap, elevated levels of plasma lactate, and low urinary pH values [12,51,52,80]. Insulin resistance, in addition to being involved in the etiology of type 2 diabetes mellitus, is a risk factor for non-alcoholic hepatic steatosis, and recently, a study has shown a positive association between NEAP and the likelihood of developing the disease. Several studies has showed that fruit and vegetable consumption is associated with lower glycosylated haemoglobin levels, greater insulin sensitivity, and a reduced risk of type 2 diabetes mellitus [81,82,83,84]. Despite their content of base-forming nutrients, fruits and vegetables also present dietary fiber that can contribute to better glycemic control [85,86].

In addition to these clinical outcomes, the high acid load of the diet has also been studied as a risk factor for hypertension. Among the mechanisms involved is the increase of the pituitary stimulus for ACTH synthesis and the consequent production of cortisol and aldosterone [28]. Furthermore, it has been suggested that intracellular potassium reduction is compensated for by elevated sodium levels and, as a consequence, blood pressure elevation [87]. In this context, studies have showed that a higher intake of fruit, vegetables [88,89,90], and some specific nutrients (i.e., potassium and magnesium) [91,92,93] are associated with a lower hypertension risk. However, there are no studies evaluating the effect of specific dietary interventions correcting low-grade metabolic acidosis on the hypertension risk. Further studies on this topic need to be performed, especially regarding increased serum bicarbonate levels and a reduced blood pressure.

Finally, the available evidences indicate the existence of a slight, yet significant, association between diet-induced low-grade metabolic acidosis, and all-cause and cause-specific mortality. As we discussed in this review, diet-induced metabolic acidosis is linked to the increased progression and risk of many non-comunicable diseases (NCDs). Since NCDs account for 60% of global deaths [94], it is possible to think that diet-induced metabolic acidosis may be associated with higher mortality rates. In addition, the acidogenic dietary pattern is characterized by a low fruit and vegetable intake. Previous studies have shown that the consumption of these food items is associated with lower mortality rates [95,96,97,98].

## 5. Conclusions

The excessive release of acids into the bloodstream may predispose to various metabolic imbalances, such as increased mineral excretion, insulin resistance, and the stimulation of glucocorticoid hormone release. Such imbalances are associated with an increased risk of non-comunicable diseases. Taking into account the acidifying potential of the western food standard of the vast majority of countries and the increase in the incidence of chronic diseases worldwide, it is necessary to encourage public policies that stimulate the intake of fruits and vegetables in the course of life, so that there is no volubility in the acid-base balance and the risk of chronic diseases is attenuated.

## Figures and Tables

**Table 1:** Estimates of the net endogenous production of acids from dietary constituents.

Net Endogenous Acid Production (NEAP, mEq/Day)	(54.5 × Protein [g/Day]/Potassium [mg/Day])−10.2
**Potential Renal Acid Load (PRAL, mEq/day)**	(0.49 × protein [g/day]) + (0.037 × phosphorus [mg/day]) − (0.021 × potassium [mg/day]) + (0.026 × magnesium [mg/day]) + (0.013 × calcium [mg/day])
**Organic Acid (OA, mEq/day)**	PRAL + body surface area * (m^2^) × 41 (mEq/day)/1.73 (m^2^)

Note: * 0.007184 × weight^0.425^ (kg) × height^0.725^ (m^2^).

**Table 2:** Renal acid load potential of foods.

Food	PRAL (100 g)	PRAL (Portion)
**Cereals**		
White rice (cooked)	1.7	1.3 (76.9 g)
Brown rice (cooked)	5.2	8.1 (156 g)
Oat flakes	9.1	2.7 (30 g)
Granola	18.4	9.0 (48.8 g)
**Vegetables**		
Pumpkin (cooked)	−1.9	−2.1 (108 g)
Zucchini (cooked)	−0.6	−0.6 (95 g)
Chard (raw)	−2.1	−1.2 (60 g)
Watercress (raw)	−1.2	−0.1 (10 g)
Artichoke (cooked)	−0.5	−0.6 (120 g)
Curly lettuce (raw)	−3.2	−0.6 (18 g)
Sweet potato (cooked)	−1.8	−1.2 (70 g)
Potato (cooked)	−1.7	−1.2 (70 g)
Eggplant (cooked)	−0.9	−0.8 (90 g)
Beet (cooked)	−2.8	−1.1 (40 g)
Beet (raw)	−5.4	−1.7 (32 g)
Broccoli (cooked)	0.8	0.2 (20 g)
Carrot (raw)	−4.4	−1.6 (36 g)
Carrot (cooked)	−1.6	−0.8 (50 g)
Shiitake mushroom (cooked)	−0.2	−0.3 (116 g)
Kale (raw)	−2.6	−0.5 (20 g)
Kale (braised)	−1.6	−0.3 (17 g)
Cauliflower (cooked)	0.2	0.2 (100 g)
Spinach (raw)	−1.5	−0.8 (50 g)
Spinach (braised)	4	2 (50 g)
Mustard leaf (raw)	−3.2	−0.7 (22.4 g)
Cucumber (raw)	−2	−0.3 (15 g)
Red bell pepper (raw)	−2.8	−0.7 (26 g)
Radish (raw)	−4.7	−0.2 (5 g)
White cabbage (raw)	−1.5	−0.3 (20 g)
Arugula (raw)	−1.1	−0.2 (15 g)
Tomato	−1.8	−0.9 (50 g)
**Fruits**		
Avocado	−2.4	−2.2 (90 g)
Pineapple	−1.1	−0.8 (75 g)
Plum	−1.7	−0.8 (45 g)
Prune	−10.1	−4.5 (45 g)
Blackberry	−1.0	−0.4 (45 g)
Banana	−5.2	−4.2 (80 g)
Cherry	−2.9	−1.8 (62 g)
Cranberry	−0.9	−0.3 (44 g)
Apricot	−3.5	−1.9 (35 g)
Raspberry	−0.6	−0.6 (90 g)
Guava	−3.3	−5.7 (170 g)
Blackcurrant	−2.6	−1.1 (45 g)
Kiwi	−3.2	−2.4 (76 g)
Orange	−1.6	−1.3 (80 g)
Lychee	−1.7	−0.3 (20 g)
Lime	−0.4	−0.1 (29 g)
Apple	−1.8	−2.3 (130 g)
Papaya	−1.1	−3.1 (270 g)
Mango	−2.2	−1.3 (60 g)
Passion fruit	−3.4	−1.5 (45 g)
Watermelon	−0.9	−1.8 (200 g)
Melon	−3.6	−3.3 (90 g)
Strawberry	−2.2	−1.1 (48 g)
Blueberry	−0.6	−1.1 (180 g)
Peach	−1.5	−0.9 (60 g)
Pomegranate	−8.1	−22.9 (282 g)
Dry date (chuara)	−8.7	−12.2 (150 g)
Green grape	−2.4	−4.1 (170 g)
Purple grape	−1.9	−3.3 (170 g)
Dried grape (raisin)	−9.0	−3.6 (40 g)
**Beans**		
Pea	4.2	1.3 (30 g)
Bean	1.5	1.2 (80 g)
Chickpea	6.3	7.6 (120 g)
Lentil	3.1	5.0 (160 g)
**Nuts**		
Almond	22.8	1.1 (5 g)
Cashew nut	23.5	9.4 (40 g)
Brazil nut	36.4	14.6 (40 g)
Walnut	15.7	4.7 (30 g)
**Fish and sea food**		
Fresh tuna (roasted)	21.7	30.4 (140 g)
Shrimp (cooked)	21.1	23.2 (110 g)
Mackerel (roasted)	16.3	14.3 (88 g)
Lobster (cooked)	51.4	59.6 (116 g)
Oyster (cooked)	12.3	5.2 (42 g)
Wild salmon (raw)	7.7	15.3 (198 g)
Wild salmon (grilled)	9.9	15.3 (154 g)
Sardine (roasted)	32.1	27.0 (84 g)
**Meat**		
Striploin steak (grilled)	19.0	28.5 (150 g)
Beef tenderloin (grilled)	21.4	32.1 (150 g)
Flank steak (cooked)	13.9	17.1 (123 g)
Eye round (cooked)	17.4	21.4 (123 g)
Chicken leg (roasted)	14.2	7.1 (50 g)
Chicken thigh (roasted)	14.8	13.5 (91 g)
Chicken chest (roasted)	19	34.9 (184 g)
**Dairy products**		
Whole cow’s milk	3.6	8.7 (240 mL)
Low-fat cow’s milk	3.9	9.4 (240 mL)
Brie cheese	16.8	13.5 (80 g)
Mozzarella cheese	39.2	7.8 (20 g)
Parmesan cheese	56.7	8.5 (15 g)
**Egg**		
Egg (cooked)	10.4	4.7 (45 g)
**Beverages**		
Coconut water	−6.1	−12.1 (200 mL)
Coffee	−2.3	−1.2 (50 mL)
